# Supplementary material for: Application of Sebum Lipidomics to Biomarkers Discovery in Neurodegenerative Diseases
Source: Metabolites. 2021 Nov 29;11(12):819. doi: 10.3390/metabo11120819 (PMC8708591; doi:10.3390/metabo11120819)
Supplement: Supplementary file 1 [file metabolites-11-00819-s001.zip › Supplementary Figure S1 Revision.pptx]

## Slide 1
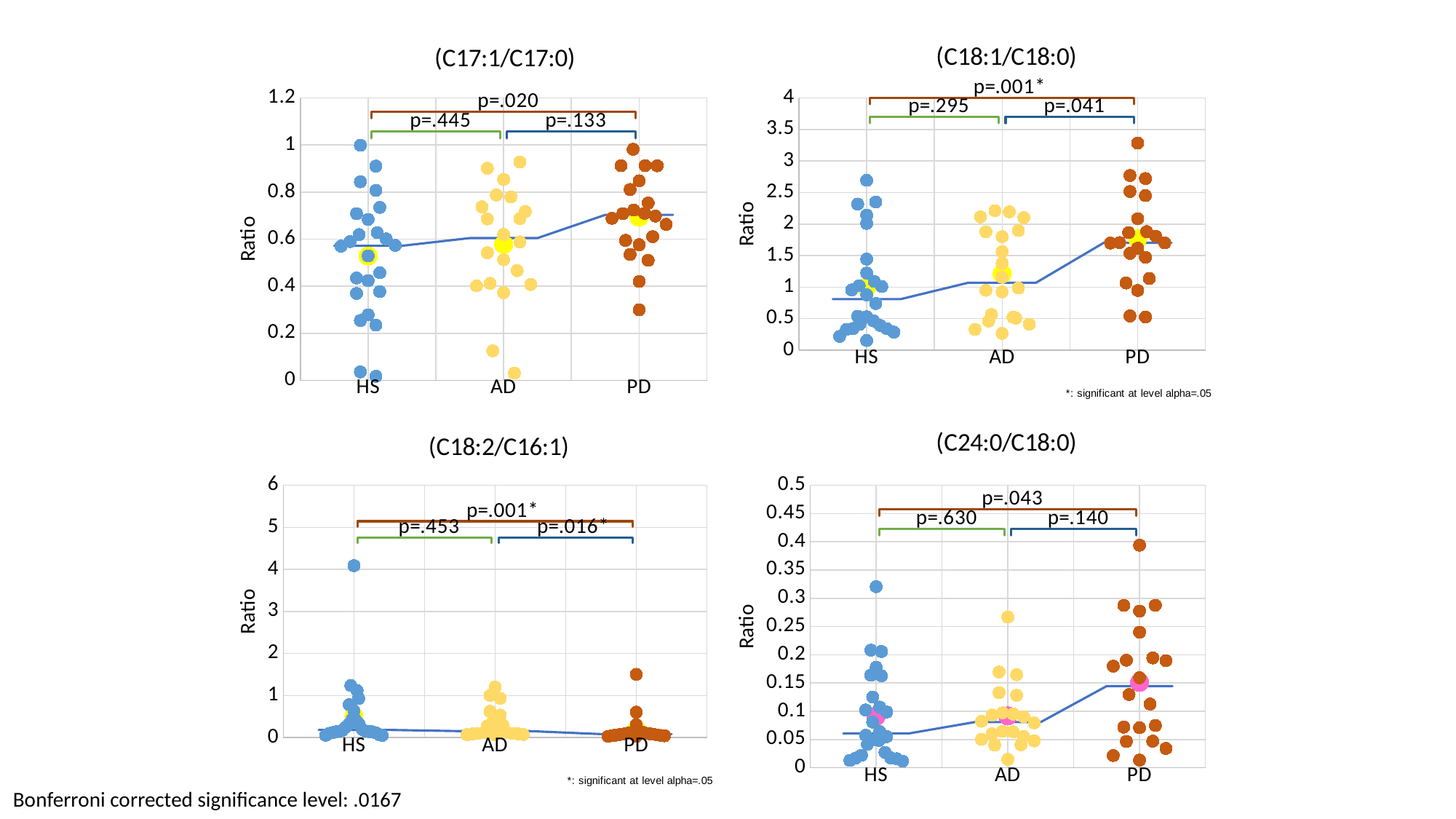

### Chart: (C17:1/C17:0)
| Category | | | | | | | | | |
|---|---|---|---|---|---|---|---|---|---|
### Chart: (C18:1/C18:0)
| Category | | | | | | | | | |
|---|---|---|---|---|---|---|---|---|---|
### Chart: (C18:2/C16:1)
| Category | | | | | | | | | |
|---|---|---|---|---|---|---|---|---|---|
### Chart: (C24:0/C18:0)
| Category | | | | | | | | | |
|---|---|---|---|---|---|---|---|---|---|Bonferroni corrected significance level: .0167
