# Supplementary figures and images for: Application of Sebum Lipidomics to Biomarkers Discovery in Neurodegenerative Diseases
Source: Metabolites. 2021 Nov 29;11(12):819. doi: 10.3390/metabo11120819 (PMC8708591; doi:10.3390/metabo11120819)

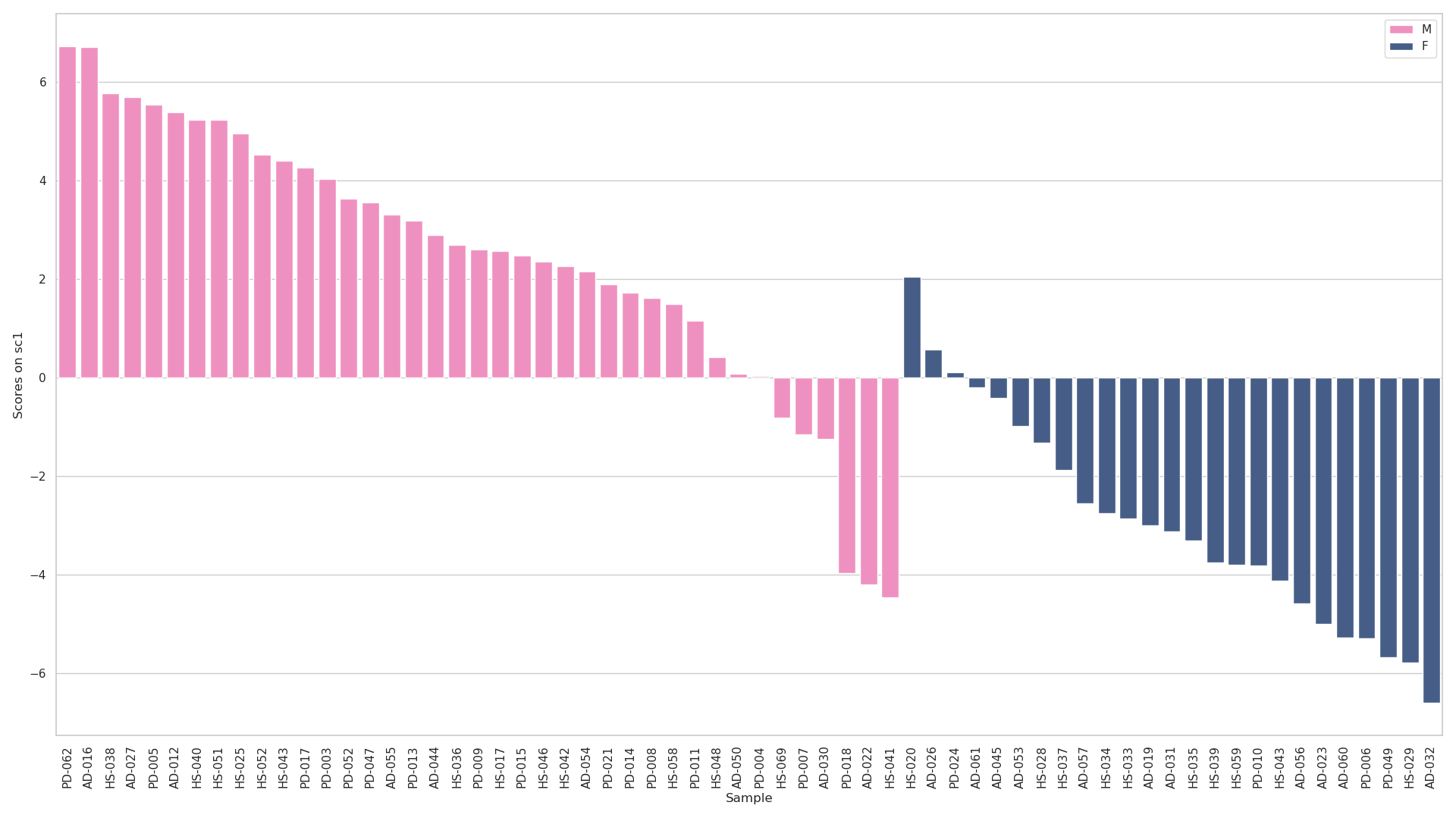

Supplement: Supplementary file 1 [file metabolites-11-00819-s001.zip › Supplementary Figure S3 (a) Scores_Gender_SC1.png]

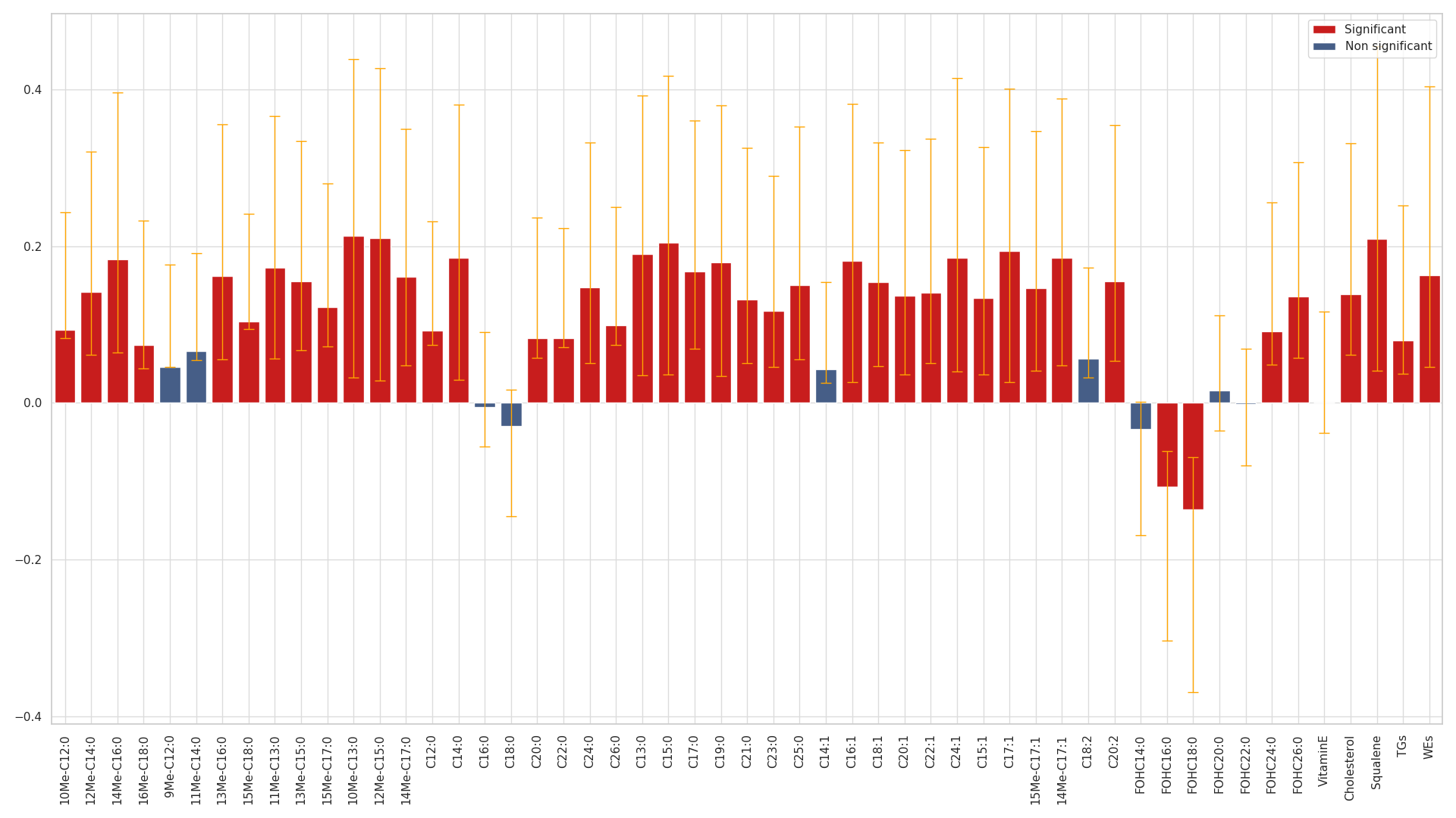

Supplement: Supplementary file 1 [file metabolites-11-00819-s001.zip › Supplementary Figure S3 (b) Loadings_Gender_SC1.png]

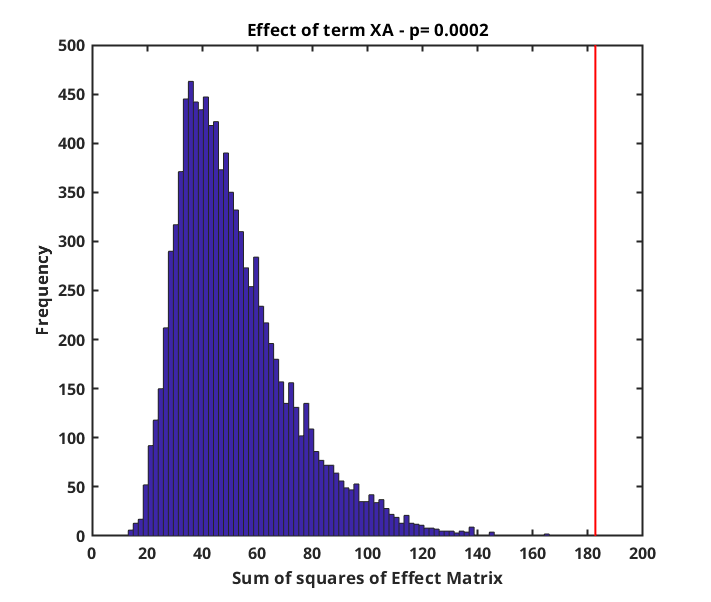

Supplement: Supplementary file 1 [file metabolites-11-00819-s001.zip › Supplementary Figure S4 (a) Females Significance_Group.png]

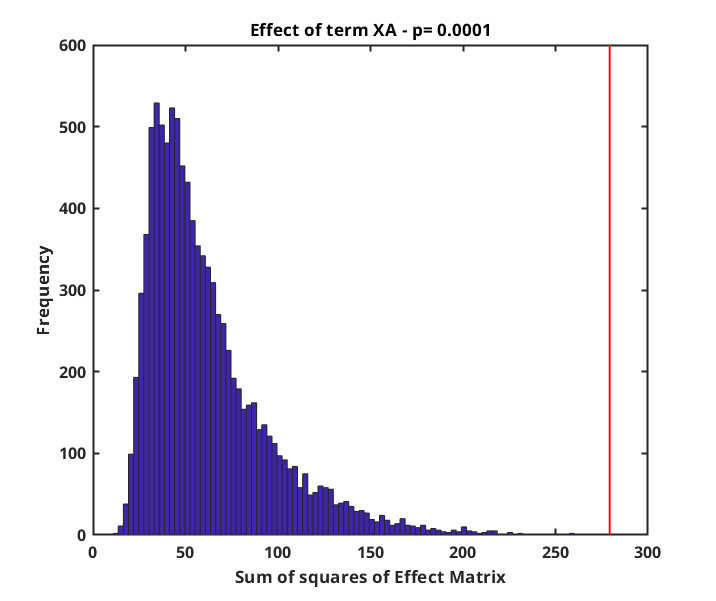

Supplement: Supplementary file 1 [file metabolites-11-00819-s001.zip › Supplementary Figure S4 (b) Males Significance_Group.png]
